# An evaluation of the population characteristics, semen quality, and utilization status of autologous sperm cryopreservation and fertility preservation in for 662 patients: a 6-year monocentric retrospective study

**DOI:** 10.1186/s12610-022-00169-5

**Published:** 2022-11-03

**Authors:** Wenhao Tang, Chenyao Deng, Jiangman Gao, Senlin Tian, Nan Wei, Bin Li, Jianfei Song, Liang Zhang, Han Wu, Hui Jiang

**Affiliations:** 1 Department of Urology, Peking University Third Hospital, Haidian District, 49 North Garden Road, Beijing, 100191 China; 2 Department of Reproductive Medicine Center, Peking University Third Hospital, Haidian District, 49 North Garden Road, Beijing, 100191 China; 3 Department of Andrology, Peking University Third Hospital, Haidian District, 49 North Garden Road, Beijing, 100191 China; 4 Department of Human Sperm Bank, Peking University Third Hospital, Haidian District, 49 North Garden Road, Beijing, 100191 China

**Keywords:** Cancer, Fertility preservation, Male, Onco-fertility, Semen cryopreservation, Sperm banking, Cancer, Préservation de la fertilité, Homme, Onco-fertilité, Cryoconservation des spermatozoïdes, Banque de sperme

## Abstract

**Background:**

Sperm cryopreservation is an effective method of fertility preservation for disease-related and social sperm freezing. In total, 662 subjects (range: 15–65 years-of-age; mean: 33.49 ± 8.79 years-of-age) were included in this study to investigate the population characteristics, semen quality, and usage of autologous sperm preservation patients in Beijing. Of these, 351 were cancer patients (53.02%, 31.14 ± 7.32 years-of-age) and 311 were non-cancer patients (46.98%, 36.14 ± 9.54 years-of-age).

**Results:**

We found that the number of preservation cases increased steadily from 2015 to 2019; 89.73% of these had a bachelor's degree or above; 54.83%, 41.54%, and 3.63% were single, married, and divorced, respectively. The cases of cancers and oligozoospermia accounted for 71.30% of all patients; therefore, most patients required fertility preservation due to disease. The cancer group had a significantly lower sperm concentration, rate of progressive sperm after the frozen-thawed test, total progressive motility sperm count after the frozen-thawed test, and recovery rate of progressive motile sperm (RRPM) than the non-cancer group (all *P* < 0.05). Sperm count-related parameters were significantly affected by testicular cancer, while sperm motility-related parameters and RRPM were significantly affected by leukemia. The utilization rate of preserved sperm was 6.34% after 6 to 78 months of follow-up. In terms of fresh or frozen embryo transfer, the clinical pregnancy rate was 56.76% or 50.00%, and the live birth rate was 24.32% or 21.43%, respectively.

**Conclusion:**

The need for autologous sperm preservation was dominated by patients with diseases, followed by the need for social sperm freezing. Tumors had a major negative impact on semen quality, and the usage rates of stored semen were at lower level compared to the number of sperm cryopreservation. Medical staff and patients should pay attention to both cognition-action consistency and cost-effectiveness in fertility preservation.

## Résumé

**Contexte:**

La cryoconservation des spermatozoïdes est une méthode efficace de préservation de la fertilité pour la congélation des spermatozoïdes liée à des causes médicales et aux demandes sociétales. Au total, 662 hommes (entre 15 et 65 ans; moyenne: 33,5 ± 8,8 ans) ont été inclus dans cette étude pour évaluer les caractéristiques de la population, la qualité du sperme et l’utilisation de la préservation autologue de spermatozoïdes réalisée par des patients à Beijing. Parmi ceux-ci, 351 étaient des patients atteints de cancer (53%; 31,1 ± 7,3 ans) et 311 des patients non atteints de cancer (47%; 36,1 ± 9,5 ans).

**Résultats:**

Nous avons constaté que le nombre de cas de conservation a augmenté régulièrement de 2015 à 2019; 89,7% d’entre eux avaient un baccalauréat ou plus; 54,8%, 41,5% et 3,6% étaient respectivement célibataires, mariés, ou divorcés. Les cas de cancer et ceux d’oligozoospermie représentaient 71,3% de tous les patients; par conséquent, la plupart des patients avaient besoin d’une préservation de la fertilité pour raison de maladie. Le groupe des hommes atteints de cancer avait significativement une plus faible concentration de spermatozoïdes, un plus faible taux de spermatozoïdes progressifs après le test de congelation-décongelation, un plus faible nombre total de spermatozoïdes de motilité progressive après le test de congelation-décongelation, et un plus faible taux de récupération de spermatozoïdes mobiles progressifs (TRMP) que le groupe de patients non atteints de cancer (tous *p* < 0,05). Les paramètres liés au nombre de spermatozoïdes ont été significativement affectés par le cancer du testicule, tandis que les paramètres liés à la mobilité des spermatozoïdes et le taux de récupération de spermatozoïdes mobiles progressifs ont été significativement affectés par la leucémie. Le taux d’utilisation des spermatozoïdes conservés était de 6,3% après 6 à 78 mois de suivi. En ce qui concerne le transfert d’embryons frais et congelés, le taux de grossesse clinique était respectivement de 56,8% et 50,0%, et le taux de naissances vivantes respectivement de 24,3% et 21,4%.

**Conclusions:**

Le besoin de conservation autologue des spermatozoïdes était dominé par les patients atteints de maladies, suivi par le besoin social de congélation des spermatozoïdes. Les tumeurs ont eu un impact négatif majeur sur la qualité du sperme, et le taux d’utilisation des spermatozoïdes stockés était à un niveau inférieur à celui du nombre de cryoconservation des spermatozoïdes. Le personnel médical et les patients doivent prêter attention à la fois à la cohérence cognition-action et à la rentabilité de la préservation de la fertilité.

## Background

As an effective modality of fertility preservation related to a range of diseases and social factors, sperm cryopreservation can be used not only for the uncomplicated aim of fertility preservation, but also for the patients of reproductive age who have certain diseases, including tumors, who require treatment that may damage the reproductive system, thus preventing the production of biological offspring. The survival rate of tumor patients has risen steadily over the last 30 years due to continuous breakthroughs in tumor diagnostics and treatment technology. The 5-year survival rate of young cancer patients at present stands at 80% [1, 2]. Reproductive health is a common concern for the survivors of male children, adolescents, and young adults who have experienced cancer. Chemotherapy medications, testicular radiotherapy, surgery, and radiotherapy of the genitourinary system/lower spinal cord segment/hypothalamic-pituitary region are all risk factors for reproductive side effects, resulting in impaired spermatogenesis, testosterone secretion insufficiency, and sexual dysfunction [3]. Germ cells are less resistant to the cytotoxic damage caused by chemotherapy and radiotherapy, and some male patients may experience infertility, oligozoospermia, or even persistent azoospermia, thus resulting in varying degrees of impaired fertility. Research involving a rodent model previously demonstrated that toxic exposure to genetic material induced chromosomal aberrations in male germ cells, which could be passed on to offspring and impact their development, fertility, and health [4]. For this group of patients, sperm cryopreservation is an ideal fertility preservation alternative to adopt prior to gonadotoxic therapy.

According to the existing literature, there are still limitations that the related knowledge mastered by the general population, patients and medical staff with regards to autologous sperm preservation, as well as the urgency of treatment for malignant tumors. Coupled with the financial constraints of patients, these factors have resulted in a low rate of autologous sperm preservation. Furthermore, there is a notable lack of detailed survey data relating to the potential population in need of autologous sperm preservation [5–9]. In the present study, we retrospectively collected data from our Human Sperm Bank over the past 6 years relating to patients who adopted the self-preservation of sperm to better understand the population characteristics, semen quality, and usage in Beijing. We also aimed to provide a foundation for future clinical work relating to autologous sperm preservation and targeted population-based scientific education.

## Material and methods

### Subjects

Six hundred and sixty-two patients who underwent autologous sperm preservation were identified from our Human Sperm Bank (Peking University Third Hospital, Beijing, China) between September 2015 and August 2021 and included in this study. For each patient, we collated information relating to current medical history, past medical history, physical examination, laboratory and ancillary tests, medication and surgical history collected at the patients’ initial visit, and the assessment of semen quality. The volume of each testis was compared with the corresponding ovoid of the Prader orchiometer.

According to disease type, the patients were sorted into a cancer group and a non-cancer group. Patients with cancers had not received chemotherapy or radiotherapy prior to autologous sperm preservation, while some of the testicular cancer (TC) patients (43/101 patients, 42.6%) had undergone surgical treatment and removal of the afflicted testis. The medical staff of the Human Sperm Bank explained the standard technique of autologous sperm preservation, related costs, freezing and storage procedures, semen frozen-thawed tests, and related instructions to the subjects. The study was authorized by Peking University Third Hospital’s Medical Ethics Committee, and all study participants signed informed consent form.

### Semen examination and cryopreservation

Semen collection methods and quality assessment methods were in accordance with the *World Health Organization (WHO) Laboratory Manual for the Examination and Processing of Human Semen (5th edition)* standards [10]. When collecting semen samples, subjects were required to maintain abstinence for 2–7 days, clean the penis and hands before masturbation. Semen samples were collected in a sterile semen collection cup. These cups were subsequently placed in a 37 °C water bath for liquefaction. A full-time laboratory technician performed routine semen testing after complete liquefaction and recorded semen volume, sperm concentration (SC), and sperm motility.

All subjects underwent the semen frozen-thawed test to determine the quality of freezing prior to sperm cryopreservation. Fresh semen samples were frozen by adding a 1:1 ratio of glycerol-egg yolk-citrate (GEYC) cryoprotectant [10]. The samples were then frozen in sealable tubes with a programmed method and then transferred to liquid nitrogen for storage. Subsequently, frozen semen was removed from the liquid nitrogen and quickly thawed in a 37 °C water bath before being re-analyzed.

### Statistical analysis

SPSS 24.0 software (International Business Machines Corp, Armonk, New York, USA) was applied to analyze the data statistically. The mean and standard deviation (S.D.) were used to describe the parameters with normal distribution. To examine statistical differences between two groups, we performed the independent samples *t*-test. Analysis of variance (ANOVA) was used to compare statistical differences among several groups. *P* < 0.05 was used to determine whether differences between groups were statistically significant.

## Results

### Statistical analysis of the number of cases and trends of patients undergoing the self-preservation of sperm by year

Over the last 6 years, 701 patients showed interest in autologous sperm preservation and came to our hospital for consultation and semen testing. Thirty-nine patients (5.6%) were diagnosed with azoospermia or lacked spermatozoa with progressive motility (PR) and gave up on semen freezing; the remaining 662 patients (94.4%) successfully implemented autologous sperm preservation and were included as subjects in this study.

**Fig. 1:**
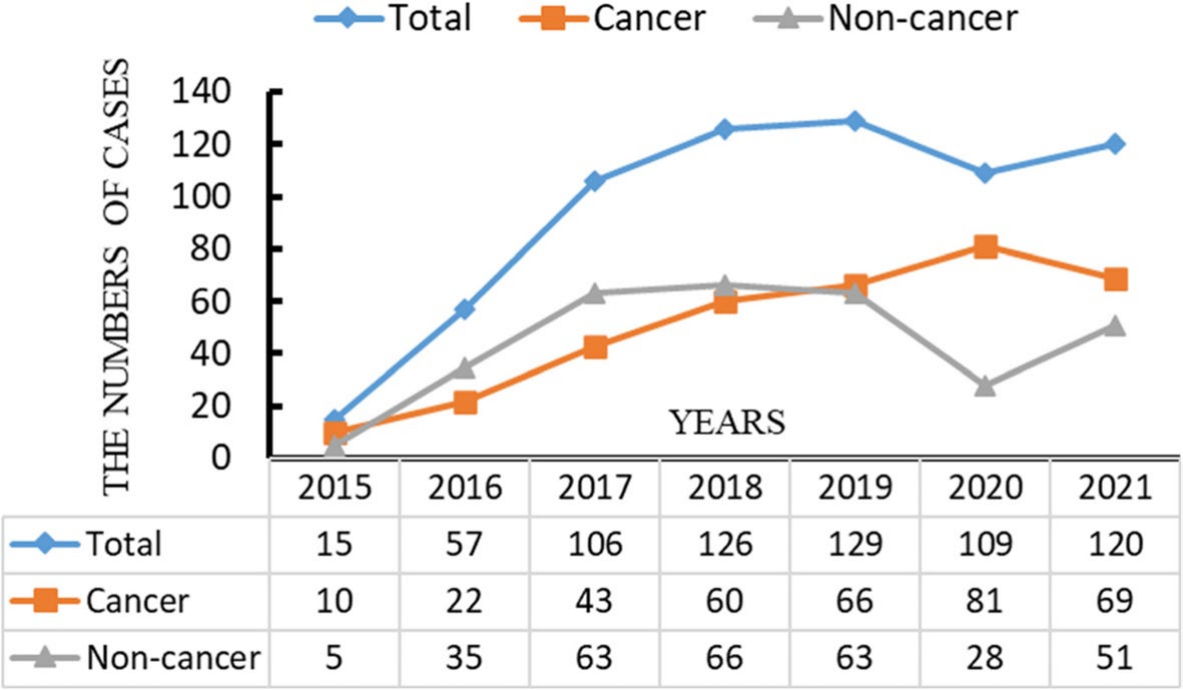
The numbers of autologous sperm preservation patients, oncology patients and non-oncology patients between 2015 to 2021 and the trend for change

Figure 1 depicts the number of autologous sperm preservation patients, cancer patients and non-cancer patients as well as trends during 2015 and 2021. The number of preservation cases out of the number of annual total cases and cancer cases increased steadily from 2015 to 2019. Owing to the impact of Coronavirus Disease 2019 (COVID-19), the number of cryopreservation cases was slightly lower in 2020 and 2021 than in 2018 and 2019; however, the number and proportion of cancer patient cases increased.

### Age and educational level

The mean age of the subjects was 33.49 ± 8.79 years. The 30–39-year-old group featured the highest number and percentage of patients (306 cases, 46.2%), followed by the 15–29-year-old group (223 cases, 33.7%). The 40–49-year-old group and the 50-year-old and above group had lower numbers than the other two groups. In teenagers aged 15 to 21 years, there were 50 cases (7.6%): 33 cancer cases and 17 non-cancer cases.

The mean age of the non-cancer patient was significantly higher than those with cancer (*P* < 0.0001). The number and proportion of patients ≥ 40 years in the non-cancer group were significantly higher than those in the cancer group (94 cases *vs.* 39 cases, 30.2% *vs.* 11.1%, *P* < 0.0001). The maximum age in the cancer group was 57 years, while the non-cancer group was 65 years; there were eight cases aged 58 to 65 years old.

In total, 594 cases (89.7%) of patients undergoing autologous sperm preservation patients had a bachelor’s degree or higher education level. The percentage of patients with a master’s degree or higher education level was significantly higher in the non-cancer group than in the cancer group (28.3% *vs.* 15.7%, *P* = 0.0001); the number and percentage of patients ≥ 40 years with a bachelor's degree or higher education level was significantly higher in the non-cancer group than in the cancer group (91 cases *vs.* 38 cases, 29.3% *vs.* 10.8%, *P* < 0.0001) (presented in Table 1).

**Table 1:** Baseline characteristics of subjects

**Parameters**		**Total** **(*n* = *662*)**	**Cancer Group**					**Non-cancer Group**					***P* value**^**a**^
			**Total (*n* = *351*)**	**15–29 years (*n* = *151*)**	**30–39 years (*n* = *161*)**	**40–49 years (*n* = *31*)**	**50–57 years (*n* = *8*)**	**Total (n = 311)**	**17–29 years (*n* = *72*)**	**30–39 years (*n* = *145*)**	**40–49 years (*n* = *65*)**	**50–65 years (*n* = *29*)**	
Age (years)		33.49 ± 8.79	31.14 ± 7.32	24.68 ± 3.58	33.94 ± 2.85	42.39 ± 2.14	53.00 ± 3.02	36.14 ± 9.54	24.42 ± 3.51	34.63 ± 2.98	43.98 ± 3.13	55.27 ± 4.66	0.000
Weight (kg)		74.70 ± 13.38	74.92 ± 14.55	72.74 ± 15.99	76.37 ± 13.63	76.71 ± 10.56	80.13 ± 13.44	74.44 ± 11.94	73.32 ± 11.55	75.06 ± 11.95	73.92 ± 11.19	75.34 ± 14.54	0.642
Height (cm)		175.78 ± 5.74	176.14 ± 5.62	175.99 ± 5.56	176.41 ± 5.75	174.68 ± 4.81	179.25 ± 6.23	175.37 ± 5.86	177.53 ± 5.40	175.13 ± 6.07	174.29 ± 6.05	173.65 ± 3.81	0.086
BMI (kg/m^2^)		24.09 ± 3.94	24.08 ± 4.11	23.42 ± 4.65	24.46 ± 3.68	25.13 ± 3.22	24.85 ± 3.30	24.10 ± 3.75	23.23 ± 3.23	24.26 ± 3.92	24.31 ± 3.33	24.97 ± 4.68	0.747
TV (ml)		25.31 ± 6.04	25.15 ± 5.91	25.18 ± 6.27	25.16 ± 5.84	25.19 ± 5.31	24.00 ± 0.00	25.59 ± 6.02	24.25 ± 7.79	25.40 ± 5.35	26.43 ± 4.83	27.97 ± 5.83	0.342
Education status	~ High school n (%)	68(10.3)	42(12.0)	33(21.9)	8(5.0)	1(3.2)	0(0)	26(8.36)	17(23.6)	6(4.1)	2(3.1)	1(3.5)	0.0003
	Bachelor n (%)	451(68.1)	254(72.4)	111(73.5)	118(73.3)	18(58.1)	7(87.5)	197(63.3)	48(66.7)	99(68.3)	34(52.3)	16(55.2)	
	Master ~ n (%)	143(21.6)	55(15.7)	7(4.6)	35(21.7)	12(38.7)	1(12.5)	88(28.3)	7(9.7)	40(27.6)	29(44.6)	12(41.4)	
Marrige status	Married n (%)	275(41.5)	120(34.2)	10(6.6)	85(52.8)	18(58.1)	7(87.5)	155(49.8)	7(9.7)	86(59.3)	43(66.2)	19(65.5)	< 0.0001
	Divorce n (%)	24(3.6)	6(1.7)	0(0)	1(0.6)	5(16.1)	0(0)	18(5.8)	0(0)	6(4.1)	8(12.3)	4(13.8)	
	Single n (%)	363(54.9)	225(64.1)	141(93.4)	75(46.6)	8(25.8)	1(12.5)	138(44.4)	65(90.3)	53(36.6)	14(21.5)	6(20.7)	
Had children before n (%)		76(11.5)	30(8.6)	2(1.3)	21(13.04)	5(16.1)	2(25.0)	46(14.6)	1(1.4)	12(8.3)	20(30.8)	13(44.8)	0.0120

*BMI* body mass index, *TV* the total volume of bilateral testes

Date presented as mean ± standard deviation (S.D.)

^a^The data from the cancer and non-cancer groups were compared using a *t* test on two sets of data

### Marital and childbearing status

With regards to marital status, the number and proportion of single men were significantly higher in the cancer group than in the non-cancer group (225 cases *vs.* 138 cases, 64.1% *vs.* 44.4%, *P* < 0.0001); while the number and proportion of cases who were married were significantly lower in the cancer group (120 cases *vs*. 155 cases, 34.2% *vs.* 49.8%, *P* < 0.0001); in each group, the number and proportion of cases who were divorced was the lowest.

The number and percentage of cases who had children was significantly lower in the cancer group than in the non-cancer group (30 cases *vs.* 46 cases, 8.6% *vs.* 14.8%, *P* = 0.0120). With increasing age, both groups showed a progressive increase in the proportion of cases who had children in each age group (*P* = 0.0002, *P* < 0.0001). One of the characteristics of autologous sperm preservation was that the older patients who had already had children were more willing to preserve spermatozoa, because they account for a higher proportion of overall patients had children (presented in Table 1).

### Status of physical examination

There were no statistically significant differences in body mass index (BMI) between the cancer and non-cancer groups (*P* = 0.747), or among different age groups in the cancer or non-cancer groups (*P* = 0.056 or *P* = 0.115).

The mean value on the total volume of subjects’ bilateral testes was 25.31 ± 6.04 ml; there were no significant differences in terms of testicular volume between the cancer and non-cancer groups (*P* = 0.342), or between age groups within the cancer group (*P* = 0.959). In the non-cancer group, however, ANOVA revealed a statistical difference in testicular volume among age groups (*P* = 0.023); multiple comparisons also revealed a statistical difference between the 17–29 year-old group and the 50–65 year-old group (*P* = 0.025) (Table 1).

### Etiological analysis of autologous sperm preservation

Most patients required sperm freezing to preserve their fertility owing to disease. For example, 53.0% of all subjects were cancer patients who required sperm preservation before radiotherapy and chemotherapy. There were 121 patients (38.9%) with oligozoospermia, 94 patients (30.2%) who requested autologous sperm preservation owing to advanced age, 26 patients (8.4%) with high-risk occupations (such as electro-welding workers and painters), 51 patients (16.4%) with planned vasectomy, and 19 patients (6.1%) with a personal willingness to undergo sperm preservation among the 311 non-cancer group patients.

### Semen quality

According to the results of semen quality assessment before sperm freezing, 172 patients were diagnosed with oligozoospermia (total sperm count (TSC) < 39 × 10^6^ per ejaculate, 25.98%), including 93 cancer patients (14.1%) and 79 non-cancer patients (11.9%), whereas 175 patients were diagnosed with asthenozoospermia (PR < 32%, 26.4%), including 82 cancer cases (12.4%) and 93 non-cancer cases (14.1%). In addition, subjects in the cancer group also had a significantly lower SC, rate of PR sperm after the frozen-thawed test (RPFT), total PR sperm count after frozen-thawed test (TPSA), and recovery rate of PR motile sperm (RRPM) than patients in the non-cancer group (all *P* < 0.05; Table 2).

**Table 2:** The frequency distribution of semen characteristics and statistical comparisons

Parameters	Total subjects (*n* = *662*) Mean ± S.D	Cancer Group						Non-cancer Group						*P* value^a^
		**Total (*n* = *351*)** **Mean ± S.D**	**15–29 years (*n* = *151*)** **Mean ± S.D**	**30–39 years (*n* = *161*)** **Mean ± S.D**	**40–49 years (*n* = *31*)** **Mean ± S.D**	**50–57 years (*n* = *8*)** **Mean ± S.D**	***P* value**^**b**^	**Total (*n* = *311*)** **Mean ± S.D**	**17–29 years (*n* = *72*)** **Mean ± S.D**	**30–39 years (*n* = *145)*** **Mean ± S.D**	**40–49 years (*n* = *65*)** **Mean ± S.D**	**50–70 years (*n* = *29*)** **Mean ± S.D**	***P* value**^**b**^	
Semen volume (ml)	2.62 ± 1.39	2.66 ± 1.42	2.63 ± 1.44	2.77 ± 1.40	2.30 ± 1.46	2.30 ± 1.54	0.326	2.59 ± 1.35	2.56 ± 1.40	2.82 ± 1.31	2.49 ± 1.31	1.72 ± 1.10	0.001^e^	0.521
Sperm concentration (× 10^6^/ml)	59.41 ± 50.29	55.56 ± 48.52	51.08 ± 51.32	57.16 ± 46.70	74.21 ± 44.33	35.39 ± 22.85	0.059	63.75 ± 51.94	47.95 ± 40.68	55.58 ± 48.37	82.29 ± 54.72	102.30 ± 58.90	0.000^f^	0.036
Total sperm count (× 10^6^/ejaculate)	147.09 ± 153.05	142.97 ± 161.69	140.12 ± 189.74	147.58 ± 141.53	154.32 ± 124.06	60.04 ± 52.81	0.489	151.73 ± 142.80	122.31 ± 140.54	144.54 ± 136.68	196.20 ± 157.68	161.06 ± 124.98	0.019^ g^	0.463
Progressive sperm rate (%)	42.16 ± 17.45	41.64 ± 17.19	43.14 ± 16.87	40.66 ± 17.42	43.55 ± 15.31	25.68 ± 18.98	0.029^c^	42.75 ± 17.75	44.67 ± 16.51	42.89 ± 17.86	42.14 ± 17.59	38.66 ± 20.50	0.483	0.414
RPFT (%)	25.34 ± 16.18	23.87 ± 15.69	24.15 ± 16.08	24.30 ± 14.89	22.93 ± 17.54	12.16 ± 15.58	0.245	27.00 ± 16.59	26.74 ± 15.86	27.36 ± 16.36	26.52 ± 17.79	23.68 ± 18.94	0.730	0.014
TPSB (× 10^6^/ejaculate)	67.45 ± 76.46	62.07 ± 74.40	57.64 ± 77.63	66.10 ± 73.49	73.78 ± 67.55	19.26 ± 33.78	0.219	73.53 ± 78.39	63.86 ± 83.19	73.34 ± 76.68	85.89 ± 79.44	70.73 ± 72.26	0.434	0.054
TPSA (× 10^6^/ejaculate)	45.40 ± 61.47	39.65 ± 58.57	35.81 ± 64.17	43.12 ± 53.11	46.17 ± 60.52	11.88 ± 25.18	0.385	51.90 ± 64.07	43.22 ± 64.33	50.98 ± 60.29	60.94 ± 69.96	50.43 ± 67.42	0.410	0.011
RRPM (%)	56.73 ± 25.46	54.59 ± 25.84	52.77 ± 26.68	56.02 ± 24.42	48.65 ± 35.24	28.25 ± 27.43	0.021^d^	59.14 ± 24.84	57.25 ± 25.01	59.41 ± 24.93	59.42 ± 25.50	47.59 ± 33.68	0.152	0.023

*RPFT* rate of progressive sperm after frozen-thawed test, *RRPM* recovery rate of progressive motile sperm, *S.D.* standard deviation;, *TPSA* total PR sperm count of after frozen-thawed test, *TPSB* total PR sperm count of before frozen-thawed test

^a^The data from the cancer and non-cancer groups were compared using a *t* test on two sets of data

^b^ANOVA of four age groups in cancer group or non-cancer group

^c^Multiple comparison in four age groups of cancer group: 15–29 years old group, 40–49 year sold group vs. 50–57 years old group: *P* = 0.026, 0.042

^d^Multiple comparison in four age groups of cancer group: 15–29 years old group, 30–39 years old group vs. 50–57 years old group: *P* = 0.011, 0.004

^e^Multiple comparison in four age groups of non-cancer group: 15–29 years old group, 30–39 years old group, 40–49 years old group vs. 50–70 years old group: *P* = 0.021, 0.000, 0.045

^f^Multiple comparison in four age groups of non-cancer group: 15–29 years old group vs. 40–49 years old group and 50–70 years old group: *P* = 0.000 and 0.000; 30–39 years old group vs. 40–49 years old group, 50–70 years old group: *P* = 0.002, 0.000

^g^Multiple comparison in four age groups of non-cancer group: 15–29 years old group vs. 40–49 years old group: *P* = 0.013

Moreover, in the cancer group, sperm motility and RRPM differed significantly across the four age groups (*P* = 0.029, 0.021). Multiple comparisons detected low levels of sperm motility and RRPM in the 50–57 year-old group. In the non-cancer group, there were significant differences in semen volume, SC, and TSC across four age groups (*P* = 0.001, 0.000, 0.019). Multiple comparisons identified that semen volume in the 50–65 year-old group was lower and the SC was higher, while in the 40–49 year-old, the SC and TSC were higher. The data of multiple comparisons presented in Table 2.

### Semen quality of subjects with different types of cancers

The different types and numbers of cancers presented in Table 3. Before sperm preservation, 43 TC patients (43/101cases, 42.6%) had undergone removal of the afflicted testis.

**Table 3:** Frequency distribution and statistical comparison of semen parameters in different types of tumors

**Parameters**	**Testicular cancer** **(*n* = *101*)** **Mean ± S.D**	**Lymphoma** **(*n* = *67*)** **Mean ± S.D**	**Leukemia** **(*n* = *44*)** **Mean ± S.D**	**Gastroenterological malignancies** **(*n* = *35*)** **Mean ± S.D**	**Other malignancies** **(*n* = *104*)** **Mean ± S.D**	***P* value**	***P* value**^**#**^ **(Multiple comparisons)**
Age (years)	29.79 ± 5.34	29.13 ± 7.67	29.68 ± 6.85	33.91 ± 6.57	33.41 ± 8.33	0.000	TC vs. GE: 0.027; TC vs. OM: 0.003; LY vs. GE: 0.012; LY vs. OM: 0.001; LE vs. OM: 0.030
TV (ml)	20.67 ± 7.44	26.45 ± 4.61	27.11 ± 3.21	27.03 ± 3.81	27.18 ± 3.78	0.000	TC vs. LY, LE, GE, OM: all 0.000
Semen volume (ml)	2.62 ± 1.13	2.71 ± 1.79	2.76 ± 1.39	2.75 ± 1.58	2.58 ± 1.40	0.937	all > 0.05
Sperm concentration (× 10^6^/ml)	31.56 ± 28.61	65.19 ± 48.22	50.13 ± 43.26	79.09 ± 71.66	67.04 ± 47.76	0.000	TC vs. LY, GE, OM: all 0.000
Total sperm count (× 10^6^/ejaculate)	79.27 ± 85.15	180.86 ± 201.80	139.05 ± 142.70	205.10 ± 247.54	161.18 ± 142.86	0.000	TC vs. LY, GE, OM: 0.000, 0.000, 0.002
Progressive sperm rate (%)	40.82 ± 16.49	46.08 ± 18.33	31.39 ± 16.88	40.00 ± 15.54	44.46 ± 16.09	0.000	LE vs. TC, LY, OM: 0.016, 0.000, 0.000
RPFT (%)	23.48 ± 13.92	26.24 ± 18.02	15.33 ± 13.88	24.43 ± 14.51	25.87 ± 15.88	0.004	LE vs. TC, LY, OM: 0.040, 0.004, 0.003
TPSB (× 10^6^/ejaculate)	34.56 ± 41.11	77.87 ± 73.90	47.75 ± 54.31	87.37 ± 115.70	76.15 ± 81.30	0.000	TC vs. LY, GE, OM: 0.001, 0.002, 0.000
TPSA (× 10^6^/ejaculate)	22.10 ± 30.73	46.06 ± 53.36	23.85 ± 30.32	62.93 ± 105.86	50.97 ± 63.21	0.000	TC vs. GE, OM: all 0.003
RRPM (%)	55.57 ± 24.88	55.49 ± 25.15	39.43 ± 30.73	61.94 ± 25.16	52.78 ± 26.79	0.002	LE vs. TC, LY, GE, OM: 0.007, 0.015, 0.002, 0.040

*GE* gastroenterological malignancies, *LE* leukemia, *LY* Lymphoma, *OM* other malignancies, *RPFT* rate of progressive sperm after frozen-thawed test, *RRPM* recovery rate of progressive motile sperm, *TC* testicular cancer, *TPSA* total PR sperm count of after frozen-thawed test, *TPSB* total PR sperm count of before frozen-thawed test, *TV* the total volume of bilateral testes

^#^The data were compared using a *t* test on two sets of data

ANOVA showed that a number of semen parameters, such as SC, TSC, motility (PR), RPFT, the total PR sperm count before the frozen-thawed test (TPSB), TPSA, and RRPM, all differed significantly across different types of cancer (all *P* < 0.01); semen volume did not differ significantly across different cancers. Analysis showed that SC, TSC, and TPSB were significantly lower in TC patients than in patients with lymphoma, gastrointestinal cancers, and other types of cancer, and that TPSA was significantly lower in TC patients than in patients with gastrointestinal cancers and other cancer types, thus indicating that TC may have a more significant impact on sperm count-related parameters. Both PR and RPFT were significantly lower in leukemia patients than in patients with TC, lymphomas, and other cancer types. Moreover, RRPM was significantly lower in leukemia patients than in patients with TC, lymphomas, gastrointestinal cancers, and other cancer types, thus indicating that leukemia might have a more significant effect on sperm motility-related parameters and RRPM (Table 3).

### The use of preserved spermatozoa and assisted reproductive technology (ART) outcomes

A total of 42 subjects, with a semen utilization rate of 6.3%, used their preserved semen to perform ARTs during 6 to 78 months of follow-up; the relevant characteristics of these patients are shown in Table 4.

**Table 4:** Clinical data of 42 couples who had used preserved semen

Contents	Parameters	Mean ± S.D
Female (*n* = *42*)	Age (years)	33.05 ± 4.42
	Duration of infertility (years)	2.65 ± 2.30
	BMI (kg/m^2^)	20.77 ± 2.62
	FSH (IU/liter)	7.00 ± 3.70
	LH (IU/liter)	3.61 ± 2.30
	E2 (pg/ml)	622.73 ± 2100.60
	TT (nmol/liter)	0.82 ± 0.42
	AMH (ng/ml)	3.23 ± 3.88
	AFC (*n*)	10.44 ± 4.34
Male (*n* = *42*)	Age(years)	35.41 ± 7.38
	BMI(kg/m^2^)	24.99 ± 4.01
	Semen volume (ml)	2.72 ± 1.73
	Sperm concentration (10^6^/ml)	46.56 ± 45.27
	PR (%)	27.41 ± 18.67
	NP (%)	4.50 ± 5.35
Embryonic development in IVF (*n* = *42*)	Oocytes (*n*)	12.10 ± 6.96
	2PN Fertilization number (*n*)	6.49 ± 4.12
	Number of Good quality embryos (*n*)	3.40 ± 2.37

*2PN Fertilization number* 2 pronuclei fertilization number, *AFC* antral follicle count, *AMH* anti-Müllerian hormone, *BMI* body mass index, *E2* estrogen, *FSH* follicle stimulating hormone, *IVF* in-vitro fertilization, *LH* luteinizing hormone, *NP* non-progressive motility, *PR* progressive motility, *S.D.* standard deviation, *TT* testosterone

In a total of 37 cycles transferred fresh embryos, the clinical pregnancy rate was 56.8% and the live birth rate was 24.3%. In a total of 14 cycles transferred frozen embryos, the clinical pregnancy rate was 50.0% and the live birth rate was 21.4%. In addition, six cycles had no available embryo development. There were 12 live births and healthy fetuses (12/42 cases, 28.6%), as well as one spontaneous abortion.

## Discussion

Since the opening of our human sperm bank (hereinafter referred to as our bank) in 2015, the total number of patients undergoing autologous sperm cryopreservation and fertility preservation, along with the number of cancer patients, has gradually increased from the years 2015 to 2019. This is related to the continual improvement of the cognition level and the acceptance of autologous sperm preservation among medical staff and the common population, as well as the rigid demands of patients for autologous sperm preservation. In particular, cancer patients have a higher demand for fertility preservation [6, 9, 11]. The literature [12] reported that only 75.0% of tumor patients received fertility preservation counseling, and 33.1% of patients completed sperm freezing. Some patients with tumors did not have their spermatozoa frozen for a variety of reasons, including a lack of knowledge about the risk of infertility, the limited availability of counseling services, and being afraid of delaying treatment [12]. Furthermore, 57.8% of male patients failed to discuss fertility preservation with the doctors prior to tumor treatment [9]. However, 87% of testicular tumor survivors were willing to preserve their spermatozoa by freezing [13]. Although the COVID-19 pandemic had a significant impact on the medical environment, the numbers of patients undergoing autologous sperm cryopreservation in our bank was still close to the numbers recorded for 2017–2019, moreover, 74.3% and 57.5% of annual cases was cancer patients in 2020 and 2021. A range of factors influenced whether sperm banking was offered to patients and taken up, such as counseling time, marriage state, patient age, disease stage, prior status of the birthed children, institutional practices, and the cost of sperm cryopreservation [14].

Our current findings revealed that the mean age of the subjects was 33.49 ± 8.79 years, with the majority of subjects aged between 15 and 39 years (79.9%), possibly indicating a greater need for fertility preservation in young men during their reproductive age. There were more single subjects than married or divorced subjects, and single men were more willing to undergo autologous sperm preservation than married or divorced men. One of the driving forces behind the urgency to preserve fertility in tumor patients is that they are younger, are more likely to be single, and have a lower proportion of children who have already been born. In addition, the older subjects whose partners had already given birth also had a high desire to preserve their spermatozoa, because they account for a higher proportion of overall patients had children. By contrast, the non-cancer group showed a significantly higher mean age, higher number and proportion of patients ≥ 40 years-of-age than the cancer group. Patients undergoing autologous sperm preservation had a higher level of education; 89.7% had a bachelor's degree or higher education level. The percentage of those with a master's degree or higher education level in the non-cancer group, and the education level of those ≥ 40 years-of-age was higher than that in the cancer group, thus suggesting that education level facilitated patients to improve their acceptance and cognition of autologous sperm preservation. In addition, the fertility preservation of non-cancer patients was associated with increased levels of initiative and unforced desire, a greater degree of motivation, and a higher level of education. Several domestic and international studies support these viewpoints [6, 12, 15]. Previously, only 6.5% of fertility-preserving cancer patients were reported to be teenagers, and the proportion of sperm freezing was lower among cancer patients aged 14 to 21 years. However, beginning semen freezing at the age of 12 was already feasible. There were no significant differences in semen quality between age groups and semen parameters were comparable to those of adults, implying that even the youngest patients should consider having their spermatozoa frozen for potential benefit and future need [16–18]. In China, adolescents aged 15 to 21 years will not have reached the legal age for marriage; we had 50 cases (7.6%) of semen preservation in this age group. This was beneficial because fertility was preserved before treatment and provided protection for the birth of biological offspring after marriage. There is a clear need for further basic and clinical research into the application of fertility preservation in unmarried and childless adolescents.

During our bank’s 6 years of operation, the percentage of tumor patients has remained between 38.6% and 74.3%, thus implying that tumor patients have a high demand for sperm cryopreservation and fertility preservation. This disease was the driving force behind most patient requests for autologous preservation. This technique allows us to preserve fertility when damage to the gametes is expected to occur. Our cancer group had 351 patients, and the non-cancer group had 121 cases of oligozoospermia, accounting for 71.3% of all patients. Zhou et al. [6] reported that tumors were the most common etiology in autologous sperm preservation (55.8%). Another report indicated that 59.2% of patients preserved sperm because of disease, and that the main etiologies requiring fertility preservation before radiotherapy and chemotherapy were TC, lymphomas, and colon cancer [15]. At the point of a definite tumor diagnosis, 96.5% of male patients were still fertile; 38% and 26.8% had a desire to have children at that time and after 2 years, respectively [9]. Older age, high-risk occupations, planned vasectomy, and personal initiative were other reasons for semen preservation in the patients in our study. Autologous sperm preservation for planned vasectomy deserves further attention, after all, the most common contraceptive methods include condoms, intrauterine devices, and oral contraceptives in the couples of reproductive stages nowadays. Pennings et al. [19] found that advanced paternal age was strongly associated with reduced fertility and an increased genetic risk in offspring, and that freezing spermatozoa at a young age was one way to avoid these outcomes. Similar to socially-factored ovum freezing, socially-factored sperm freezing could be developed to some extent. The main difference between these two techniques is that the latter is less concerned with fertility preservation and more concerned with avoiding increased genetic risk in offspring. Delaying childbearing for non-medical reasons, social, and cultural reasons has become an emerging trend at home and abroad, and age-dependent reductions in fertility are known to have a significant impact on the success of delivery. Age and genetic factors (included epigenetic factors) have a significant impact on the integrity of sperm. Men > 40 years-of-age have a higher risk of passing on age-related mutations to their offspring. Gromoll et al. [20] suggested that men who delay childbearing could opt for sperm cryopreservation.

We found that 26.0% and 26.4% of our subjects suffered from oligozoospermia and asthenozoospermia, respectively. Furthermore, the cancer group had lower SC, RPFT, TPSA, and RRPM than the non-cancer group. In addition, the 50–57 years subgroup of the cancer group exhibited lower sperm motility and frozen-thawed recovery rates. Because the cancer and non-cancer groups showed no statistically significant differences in BMI or testicular volume, these two factors did not interfere with the semen parameter results. These findings agreed with those reported by Zhou et al. [6]. In other studies [8, 15, 18], 83.1% of patients undergoing autologous sperm preservation exhibited normal semen parameters; oligozoospermia was the most common etiology, and the sperm count of cancer and non-cancer patients was lower than healthy controls; these data were similar to our present findings. In non-cancer patients, we found that the 50–65 year-old group had a higher SC and that the 40–49 year-old group had a higher SC and TSC; this could be due to the bias of semen parameters in a specific population and the relatively small sample size of patients undergoing autologous sperm preservation.

TC, lymphomas, and hematological tumors are the most common types of tumors in patients undergoing autologous sperm preservation, according to the literature, followed by head and neck, chest, abdomen, prostate, bone and soft tissue, and other types of malignant tumors [21–25]. Our research found that 28.8% of tumor patients had TC, 19.1% had lymphoma, and 12.5% had leukemia; these findings are similar to those reported previously.

Germ cell tumors in the testicles make up 1% of all tumor types. A previous study found that TC was the most common form of cancer in men aged 15 to 40 years and that one of the main concerns for tumor survivors was fertility [14, 26]. At the time of diagnosis, 6% to 24% of TC patients had azoospermia, 50% had oligozoospermia; these patients also showed a 30% reduction in male fertility due to gonadotoxic treatment, and only 24% carried out semen preservation [27]. In the present study, we found that there were statistically significant differences in semen parameter values such as SC, TSC, motility (PR), RPFT, TPSB, TPSA, and RRPM among different tumor types and that TC had a significant effect on sperm count parameters. Leukemia had a significant effect on sperm motility-related parameters and RRPM. Previous research demonstrated that a variety of tumors had a significant and negative impact on the quality of semen and the production of sperm in young adult men, and that patients with testicular or hematological tumors had a significantly lower SC or TSC, RRPM, and TPSA; furthermore, the lowest PR percentages were found in patients with leukemia and brain tumors [7, 11, 21, 22, 28, 29]. According to previous findings, 38.3% of tumor patients undergoing autologous sperm preservation had normal semen quality, 7.5% had azoospermia, 57.2% had severe asthenozoospermia, 22.3% had severe oligozoospermia, and patients with TC had the lowest semen parameters [30]. Reduced fertility and semen quality in patients with testicular tumors may be due to the impairment of spermatogenesis or testicular tumor-related factors (for example, the levels of β-human chorionic gonadotrophin, α-fetoprotein and lactate dehydrogenase) interfering with hypothalamic-pituitary–gonadal axis function, thus affecting testicular spermatogenesis [27]. Because testicular tumor patients were mostly treated in the urology and andrology departments, and were treated by specialized doctors, these patients received consultation relating to semen cryopreservation earlier and had a higher cognitive level of the process. Therefore, more patients underwent autologous sperm preservation; thus, data were richer and more detailed.

Several previous studies [21, 26, 31] reported lymphoma-related data: the semen parameters in patients with TC were the worst prior to chemotherapy; this was followed by Hodgkin’s lymphoma (HL). The sperm quality of patients with TC and non-Hodgkin’s lymphoma (nHL) were lower than the normal population, and less than 60% of nHL patients met the normal reference standards for semen quality [31]. Paoli et al. found that semen parameters were normal in 75% of patients with HL and concluded that HL itself may not be the main cause of impaired spermatogenesis; rather, the treatment was the main influence and the severity of impairment depended on the type of regimen and the number of cycles [32]. Lymphoma was commonly seen in the patients attending our bank (19.1%); the mean age of these patients was younger; this group is worth investigating further.

Over a follow-up period of 6 to 78 months in our bank, the proportion of subjects who used their preserved semen to undertake ART (in-vitro fertilization/intracytoplasmic sperm injection, IVF/ICSI) was 6.34%. Most previous studies reported that the use of preserved sperm ranged from 3.0% to 11.9% [7, 8, 15, 16, 23, 28, 30, 31, 33, 34], with a few studies reporting rates as high as 17.2% to 23.2% [35]. Researchers agree that the use of cryopreserved spermatozoa was low, and that sperm freezing was an important part of the comprehensive suite of treatments for men undergoing gonadotoxic therapy; however, this strategy is under-utilized. A recent study hypothesized that the lack of reporting on fertility outcomes of sperm cryopreservation was the reason for under-utilization and that short follow-up periods often led to a lack of reporting on fertility outcomes; these authors suggested that pooling this information would be beneficial for semen utilization [8]. A short follow-up time is often one of the reasons for low semen utilization and the lack of reporting for reproductive outcomes. A range of published literature [23, 31, 34, 35] indicated that the duration of follow-up varied from 1 month to 26 years, and that the usual follow-up period was 3 to 10 years; our present findings concur with these previous data.

IVF and ICSI are the most commonly used forms of ART following the cryopreservation of sperm. It has also been suggested that ICSI may optimize the chances of pregnancy, regardless of the fertility status of the female partner, given the decreased quality of the frozen-thawed test for semen frozen over a long period of time [21, 25]. Clinical pregnancy rates for ART cycles ranged from 45.8% to 82% while live birth rates ranged from 35 to 82%, thus demonstrating the achievement of fertility and safe pregnancies with frozen-thawed sperm [36]. Depalo et al*.* [22] concluded that the rates of motility, vitality, and fertilization of thawed semen were significantly lower in patients with testicular tumors and lymphomas than other tumors (35.4% and 50% *vs.* 71.4%), while there were no differences in the ovum cleavage rate and implantation rates. In terms of fresh or frozen embryo transfer, our clinical pregnancy rate (56.8% or 50.0%) was similar to previously published data, while our live birth rate (24.3% or 21.4%) was lower than that reported in the literature.

## Conclusions

The number of patients undergoing autologous sperm cryopreservation and fertility preservation is increasing annually and is particularly high in young men of reproductive age. Patients in the cancer group were younger, more likely to be single, and were more likely to be childless. Tumors and oligozoospermia were the main etiological factors in 71.3% of patients. In conclusion, the need for autologous sperm preservation was dominated by patients with diseases, followed by the need for social sperm freezing. Tumors had a major negative impact on semen quality, and the usage rates of stored semen were at lower level compared to the number of sperm cryopreservation. Medical staff and patients should pay attention to both cognition-action consistency and cost-effectiveness in fertility preservation.

## Data Availability

All data are available from the authors on reasonable request.

## Abbreviations

ANOVA: Analysis of variance
ART: Assisted reproduction technique
BMI: Body mass index
COVID-19: Coronavirus Disease 2019
GEYC: Glycerol-egg yolk-citrate
HL: Hodgkin's lymphoma
IVF/ICSI: In-vitro fertilization/intracytoplasmic sperm injection
nHL: Non-Hodgkin's lymphoma
RPFT: Rate of progressive sperm after the frozen-thawed test
RRPM: Recovery rate of progressive motile sperm
SC: Sperm concentration
TC: Testicular cancer; progressive motility (PR)
TPSA: Total PR sperm count after frozen-thawed test
TPSB: Total PR sperm count before the frozen-thawed test
TSC: Total sperm count
